# Psychometric validation of the Cognitive Abilities Screening Instrument using Rasch analysis in people with dementia

**DOI:** 10.1097/MD.0000000000034093

**Published:** 2023-08-11

**Authors:** Shu-Chun Lee, Ya-Chen Lee, En-Chi Chiu

**Affiliations:** a Department of Occupational Therapy, Taipei City Hospital Songde Branch, Taipei, Taiwan; b Department of Recreation and Sports Management, University of Taipei, Taipei, Taiwan; c Department of Occupational Therapy, Jen-Teh Junior College of Medicine, Nursing and Management, Miaoli, Taiwan; d Department of Occupational Therapy, College of Medical and Health Science, Asia University, Taichung, Taiwan; e Department of Long-Term Care, National Taipei University of Nursing and Health Sciences, Taipei, Taiwan.

**Keywords:** cognition, dementia, outcome assessment, Rasch analysis, unidimensionality

## Abstract

This study aimed to examine unidimensionality of the Cognitive Abilities Screening Instrument (CASI) using Rasch analysis and estimate Rasch person reliability in people with dementia. CASI data of people with dementia was collected from medical records of one general hospital in northern Taiwan. A total of 506 people with dementia were recruited from the Department of Neurology. The confirmatory factor analysis was first conducted to verify the fitness of one-factor model of the CASI. Unidimensionality was confirmed through 2 assumptions: the infit and outfit mean square were 0.5 to 1.5, and residual variance of the first principal component in principal component analysis was ≤20%. Rasch person reliability was estimated after undimensionality was supported. The results of one-factor model had shown that the Comparative Fit Index = 0.99, Tucker Lewis Index = 0.99, Root Means Square Error of Approximation = 0.015, and Standardized Root Mean Square Residual = 0.067, representing a good fit to the model. Both the infit and outfit mean square were ranged 0.87 to 1.37 and 0.86 to 1.42, respectively, and low residual variance of the first principal component (12.3%). Rasch person reliability result of 0.58 was satisfactory. The person-item map indicated the difference between item difficulty and person ability was within the acceptable limits (0.22 logits). Differential Item Function was found between −0.48 to 0.44 logits for gender, indicating the CASI functioned similarly for both genders. The 46 items of the CASI showed a unidimensional construct. The CASI had been demonstrated as a valid and reliable screening tool for assessing overall cognitive function in people with dementia, capturing their cognitive functions effectively.

## 1. Introduction

As the trend in global aging increases, the aging population experiencing dementia is rapidly growing. An estimated 47 million people worldwide are suffering from dementia.^[1]^ Progressive cognitive decline is the dominant symptom in people with dementia, including deficits in memory, attention, executive functions, and language.^[2]^ Cognitive deficits interfere in the performance of daily tasks, work, and social activities for people with dementia.^[3,4]^ Consequently, the cost of care and social welfare due to dementia places a heavy burden on caregivers and society. Determining the cognitive function of people with dementia and monitoring their cognitive status is crucial for planning health services. Therefore, a valid and reliable cognitive screening tool should be available to assess overall cognitive function in people with dementia.

The Cognitive Abilities Screening Instrument (CASI) is one of the most commonly used cognitive screening tools to assess overall cognitive function in people with dementia.^[5]^ The CASI was designed based on symptoms diagnosed as dementia and 3 cognitive screening tools: the Mini Mental State Examination, the Modified Mini-Mental State test, and the Hasegawa Dementia Screening Scale.^[6]^ The CASI has 3 features. First, it assesses overall cognitive function with 9 dimensions to offer cognitive portraits in a comprehensive manner. The test results of individual dimensions capture the advantages and disadvantages of different aspects of cognitive functions. Second, it has cross-cultural application in measuring the severity of dementia. Third, the score of the Mini Mental State Examination can be estimated from the CASI.^[7]^ Thus, the CASI is an adequate screening tool to assess overall cognitive function in people with dementia for clinicians and researchers.

Regarding psychometric properties of the CASI, it has sufficient ecological validity, convergent validity, discriminative validity, and test-retest reliability for people with dementia.^[8,9]^ The construct validity of the CASI has been evaluated using confirmatory factor analysis in previous studies. The results showed that the one-factor model (i.e., unidimensionality) was not supported in normal adults and people with Alzheimer’s disease,^[10]^ and the two-factor model was suggested in normal males.^[11]^ Unidimensionality verifies whether all items of a measure reflect a single theoretical construct.^[12]^ Aggregating the item scores in the CASI has been applied to describe the construct (i.e., overall cognitive function). The psychometric properties of a screening tool are sample dependent and should be examined in a specific sample (e.g., people with dementia).^[13]^ Hence, examining the unidimensionality of the CASI in people with dementia is necessary to determine whether clinicians and researchers can appropriately use the sum scores of the CASI to describe overall cognitive function.

To examine unidimensionality using the classical test theory (e.g., confirmatory factor analysis), the score of each item is ranked as equivalent on an ordinal-level scale, which does not consider the differences in difficulty for individual items.^[14]^ Similarly, the total score is used to describe the single construct without considering the differences in difficulty for individual items. In Rasch analysis, the Rasch model converts ordinal raw scores into interval scores using logit statistical method, thus item difficulty is ranked among all items.^[15]^ Moreover, Rasch analysis estimates Rasch person reliability, which is an index of measurement error. Rasch person reliability reflects the extent of the scores of a measure influenced by measurement error.^[12]^ A higher Rasch person reliability demonstrates that measurement error is smaller (i.e., more precise on the scores of a measure). Rasch person reliability is similar to internal consistency in classic test theory.^[16]^ For the CASI, unidimensionliaty using Rasch analysis has not been evaluated in people with dementia, limiting its utility in clinical and research settings. Therefore, the purposes of this study were to evaluate the unidimensionality using Rasch analysis of the CASI and examine the Rasch person reliability in people with dementia.

## 2. Methods

### 2.1. Participants

A retrospective study was conducted and data were collected from electronic medical records at one general hospital in northern Taiwan. The CASI was administered from January 2017 to August 2019 for people with dementia at the Department of Neurology. The data were included if they met the following criterion: patients who had been diagnosed with dementia by neurologists. The exclusion criteria were as follows: having a history of brain injury, and having a diagnosis of mental retardation or mental illness. In addition, the sample size was determined to be at least 200 participants to allow for more accurate interpretations of our study results, as recommended in the literature.^[17]^ This study was approved by the Taiwan Adventist Hospital Institutional Reviewer Board (File #: 108-E-09). Further informed consents for our study were not sought because the data utilized devoid of personal identifiers. Our study was conducted in accordance with the Declaration of Helsinki.

### 2.2. Procedure

The CASI was administered to patients by a psychologist. The data from these 3 years were collected for data analysis. One research assistant reviewed the electronic medical records, chose the eligible participants, and collected participants’ demographic information, CASI data, and the Clinical Dementia Rating (CDR).

### 2.3. Instrument

The CASI measures overall cognitive function across 9 dimensions with 46 items. The 9 dimensions (number of items, score range) are as follows: long-term memory (5 items, 0–10), short-term memory (6 items, 0–12), attention (6 items, 0–8), mental manipulation (5 items, 0–10), orientation (9 items, 0–18), abstraction and judgment (6 items, 0–12), language (6 items, 0–10), visual construction (1 item, 0–10), and list-generating fluency (1 item, 0–10). The long-term memory dimension assesses the abilities of recalling general knowledge. The short-term memory dimension assesses the abilities of holding information provided over a short time. The attention dimension assesses the abilities of repeating sentences and words. The mental manipulation dimension assesses the abilities of arithmetic. The orientation dimension assesses the abilities of orienting place, time, and age. The abstraction and judgment dimension assesses the abilities of problem solving. The language dimension assesses the abilities of naming, reading, writing, and following instructions. The visual construction dimension assesses the abilities of copying figures. The list-generating fluency dimension assesses the abilities of speaking out 4-legged animals. The sum of scores on the 9 dimensions is the total score of the CASI, ranged from 0 to 100. A greater score demonstrates better overall cognitive function.^[6]^

The CDR measures cognitive and functional impairments for people with dementia. It contains 6 domains: orientation, memory, judgment and problem solving, community affairs, home and hobbies, and personal care.^[18]^ The 5 domains (i.e., orientation, memory, judgment and problem solving, community affairs, and home and hobbies) contain 5 grades (0-0.5-1-2-3). The personal care domain is divided into 4 scoring grades (0-1-2-3). The CDR score is estimated from 6 domains, which decides the severity of dementia: 0 as healthy, 0.5 as questionable dementia, 1 as mild dementia, 2 as moderate dementia, and 3 as severe dementia.^[19]^ The CDR has sufficient reliability and validity in people with dementia.^[20]^

### 2.4. Data analysis

The confirmatory factor analysis (CFA) was first conducted to verify the fitness of one-factor model of the CASI.^[21,22]^ Among researches, different fitness indices have been reported in the studies. The current study utilized common fitness index used by many studies: Comparative Fit Index (CFI), Tucker Lewis Index (TLI), Root Means Square Error of Approximation (RMSEA), and Standardized Root Mean Square Residual (SRMSR). A model fit was indicated using a set of cutoff values: the CFI > 0.95, TLI > 0.95, RMSEA < 0.06, and SRMSR < 0.08.^[23]^

Then, the Rasch analysis was conducted to examine the unidimensionality of the CASI using a partial credit model in the WINSTEPS computer program. The infit and outfit statistics were applied to verify whether item responses fit the expectations of the unidimensional Rasch model. An item with an infit or outfit mean square < 0.5 or > 1.5 demonstrated a misfit.^[24,25]^ We deleted the misfit item and then reconducted the Rasch analysis to ensure unidimensionality. Principal component analysis (PCA) on residuals was applied to further ascertain the unidimensionality. The residual variance of the first principal component was ≤20%, demonstrating unidimensionality.^[16]^ Rasch person reliability was analyzed for the items which fitted the unidimensional Rasch model. The criteria of Rasch person reliability was ≥0.70, good reliability, and 0.40 to 0.70, acceptable reliability.^[26,27]^

Rasch modeling allows the estimation of both item difficulty and person ability for a measure.^[28]^ The person-item map displays the location of person abilities and item difficulties respectively along the same latent dimension. By examining the person-item map could illustrate differences between a person’s abilities and item difficulty. A difference greater than1 logit indicates significant mistargeting while a difference of zero characterizes a perfectly-targeted measure.

Differential Item Function (DIF) was also carried out to examine whether the different gender (male/female) had systematically different responses to particular items. The DIF contrast is the difference in the difficulty of the item between the 2 subgroups and the cutoff of a DIF > 1 logit was used to whether a notable DIF existed across gender.^[28]^

Since Rasch analysis allows to transform the ordinal scores of the measure to an interval scale, the conversion table was obtained once the best fitting model had been found. The corresponding interval-level scores in logits and raw score of ordinal scale range were obtained.^[29]^

The floor and ceiling effects were evaluated for each dimension of the CASI. Floor and ceiling effects were analyzed by the percentage of participants with the lowest and highest scores, respectively, in each dimension. A percentage ≥ 15.0% indicated noticeable floor and ceiling effects.^[30]^

## 3. Results

Data from a total of 506 people with dementia were collected. The mean age was 67.5 years. About half of the participants (50.4%) were male. Approximately one third of participants (33.6%) were scored 1 on the CDR. Table 1 provides further details on demographic characteristics.

**Table 1:** Demographic characteristics of participants (n = 506).

Characteristics and measures	
Age, mean year (SD)	67.6 (13.1)
Gender, n (%)	
Male	257 (50.8)
Female	249 (49.2)
Education, n (%)	
Elementary school and below	254 (50.2)
Middle school	80 (15.8)
High school	95 (18.8)
College and above	77 (15.2)
Diagnosis	
Alzheimer’s disease	393 (77.7)
Vascular dementia	113 (22.3)
CDR	
1	170 (33.6)
2	169 (33.4)
3	167 (33.0)
CASI dimension, mean (SD)	
Long-term memory	6.0 (3.4)
Short-term memory	7.1 (1.9)
Attention	5.5 (1.3)
Mental manipulation	5.4 (2.6)
Orientation	10.0 (5.1)
Abstraction and judgment	6.0 (2.9)
Language	7.1 (1.3)
Visual construction	5.0 (3.1)
List-generating fluency	4.8 (3.1)

CASI = Cognitive Abilities Screening Instrument, CDR = Clinical Dementia Rating, SD = standard deviation.

Applying the Modification indices by adding an Error Covariance, the results of one-factor model had shown that the Comparative Fit Index (CFI) = 0.99, Tucker Lewis Index (TLI) = 0.99, Root Means Square Error of Approximation (RMSEA) = 0.015, and Standardized Root Mean Square Residual (SRMSR) = 0.067, representing a good fit to the model.

Both the infit and outfit mean square were ranged 0.87 to 1.37 and 0.86 to 1.42, respectively, indicating an adequate fit to the model (Table 2). The residual variance of the first principal component in PCA was 12.3% for the CASI. Rasch person reliability result of 0.58 was satisfactory, which implies the acceptable internal consistency for the CASI in this sample.

**Table 2:** Item difficulty, infit and outfit statistics of the CASI (n = 506).

Dimension	Item	Item difficulty	Infit MnSq	Outfit MnSq
Long-term memory	1. General knowledge-month	−0.13	0.93	0.93
	2. General knowledge-new year	−0.25	0.98	0.98
	3. General knowledge-minute	−0.25	0.98	0.98
	4. General knowledge-sun	−0.13	0.93	0.93
	5. General knowledge-moon	−0.13	0.93	0.93
Short-term memory	6. Recall 1a	0.12	1.11	1.13
	7. Recall 1b	0.05	1.06	1.07
	8. Recall 1c	0.10	1.06	1.07
	9. Recall 2a	0.08	0.99	0.99
	10. Recall 2b	0.08	0.99	0.99
	11. Recall 2c	0.08	0.99	0.99
	12. Recall objective	0.06	1.16	1.18
Attention	13. Registration	0.14	1.03	1.03
	14. Repetition a	0.15	1.00	1.00
	15. Repetition b	0.01	1.06	1.06
Mental manipulation	16. Digit backward a	0.00	1.00	1.00
	17. Digit backward b	0.14	0.87	0.86
	18. Digit backward c	0.14	0.87	0.86
	19. Subtraction a	−0.07	1.02	1.02
	20. Subtraction b	0.08	1.02	1.02
	21. Subtraction c	0.14	0.99	0.99
	22. Subtraction d	0.16	0.99	0.99
	23. Subtraction e	0.16	0.99	0.99
Orientation	24. Age	−0.25	0.98	0.98
	25. Year	−0.68	0.92	0.91
	26. Month	0.18	0.90	0.89
	27. Date	0.15	0.92	0.91
	28. Day	−0.38	0.94	0.93
	29. Today	−0.99	0.96	0.94
	30. Space a1	0.14	0.87	0.86
	31. Space a2	0.14	0.87	0.86
	32. Space b	0.18	0.91	0.90
Abstraction and judgement	33. Similarity a	0.10	1.05	1.05
	34. Similarity b	0.12	1.09	1.09
	35. Similarity c	0.13	1.03	1.04
	36. Judgement a	0.09	0.99	0.98
	37. Judgement b	0.09	0.99	0.98
	38. Judgement c	0.09	0.99	0.98
Language	39. Read	0.05	1.09	1.09
	40. Write	0.01	1.18	1.21
	41. Name body	−0.04	1.03	1.03
	42. Name object a	0.00	1.00	1.00
	43. Name object b	0.00	1.00	1.00
	44. Follow command	0.01	1.02	1.02
Visual construction	45. Draw	0.00	1.00	1.00
List-generating fluency	46. Animal	0.15	1.37	1.42

CASI = Cognitive Abilities Screening Instrument, MnSq = mean square.

**Figure 1.**
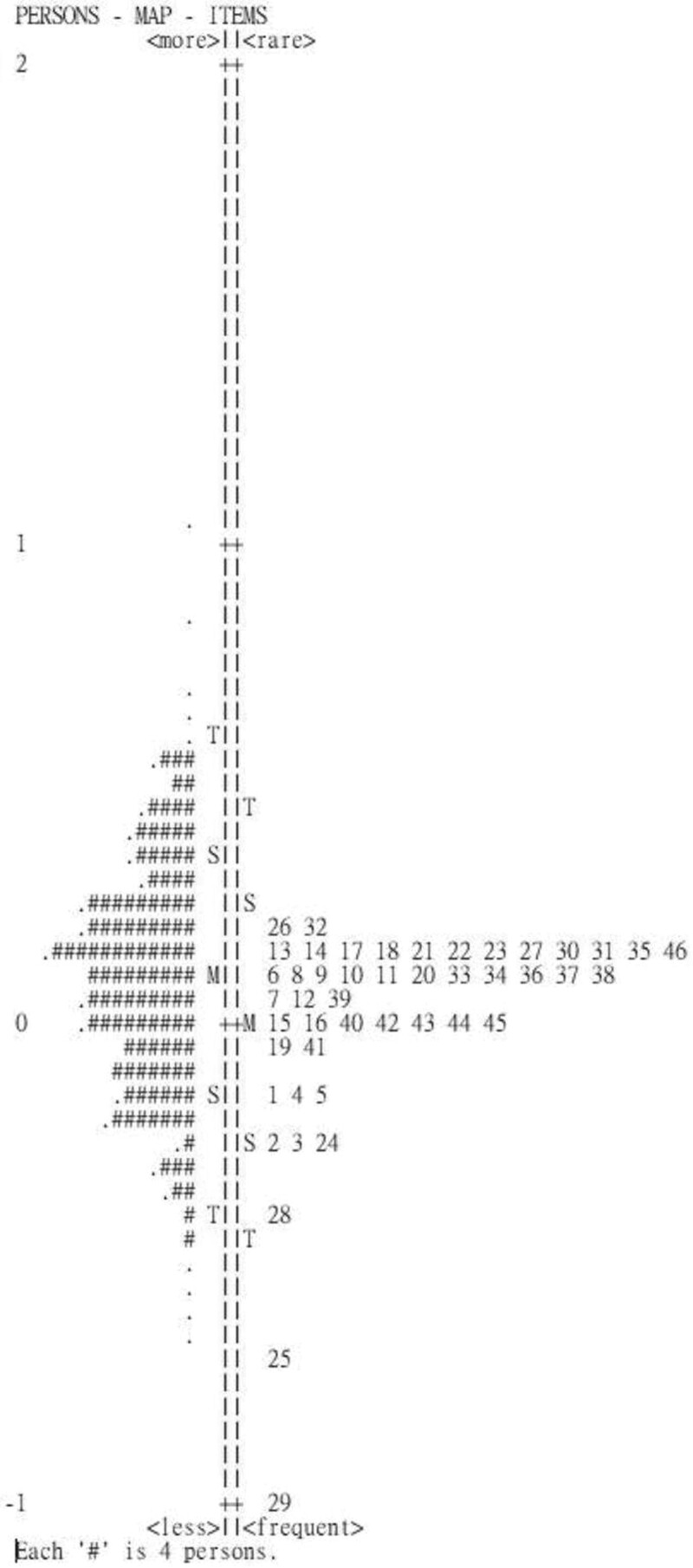
Person-item map for the CASI. Participants were represented on the left of the dashed line by the symbol “#” (which represent 4 participants) and “.” (which indicates 1 participant). On the right of dashed line were illustrated the items of CASI 46-item version with their number. Higher ability for participants and more difficult items were on the top of the figure. CASI = Cognitive Abilities Screening Instrument.

As indicated, the person-item map (Fig. 1), the difference between item difficulty and person ability was within the acceptable limits (0.22 logits). The results showed that the DIF was absent (−0.48 to 0.44 logits) for gender which suggests that the items of the CASI functioned equally for the participants, independently of their gender. Table 3 presents the conversion table of raw scores of the CASI to Rasch scale scores. It shows that the ordinal scale and corresponding interval-level scores.

**Table 3:** Conversion table from raw sum scores of the CASI to Rasch interval scores.

Raw sum score	Logit location	Interval score (0–100)
0	−2.15	0.00
1	−2.10	1.37
2	−2.05	2.74
3	−2.00	4.11
4	−1.95	5.48
5	−1.90	6.85
6	−1.85	8.22
7	−1.81	9.32
8	−1.77	10.41
9	−1.73	11.51
10	−1.69	12.60
11	−1.64	13.97
12	−1.59	15.34
13	−1.54	16.71
14	−1.49	18.08
15	−1.44	19.45
16	−1.39	20.82
17	−1.34	22.19
18	−1.29	23.56
19	−1.24	24.93
20	−1.20	26.03
21	−1.16	27.12
22	−1.12	28.22
23	−1.08	29.32
24	−1.04	30.41
25	−1.00	31.51
26	−0.97	32.33
27	−0.94	33.15
28	−0.91	33.97
29	−0.88	34.79
30	−0.85	35.62
31	−0.82	36.44
32	−0.78	37.53
33	−0.74	38.63
34	−0.70	39.73
35	−0.66	40.82
36	−0.62	41.92
37	−0.58	43.01
38	−0.54	44.11
39	−0.51	44.93
40	−0.48	45.75
41	−0.44	46.85
42	−0.40	47.95
43	−0.37	48.77
44	−0.34	49.59
45	−0.31	50.41
46	−0.28	51.23
47	−0.25	52.05
48	−0.22	52.88
49	−0.20	53.42
50	−0.17	54.25
51	−0.14	55.07
52	−0.12	55.62
53	−0.09	56.44
54	−0.07	56.98
55	−0.04	57.81
56	−0.02	58.36
57	0.01	59.18
58	0.03	59.73
59	0.06	60.55
60	0.08	60.10
61	0.11	61.92
62	0.13	62.47
63	0.16	63.29
64	0.18	63.84
65	0.20	64.38
66	0.23	65.21
67	0.25	65.75
68	0.28	66.58
69	0.31	67.40
70	0.33	67.95
71	0.36	68.77
72	0.39	69.59
73	0.41	70.14
74	0.44	70.96
75	0.47	71.78
76	0.50	72.60
77	0.53	73.42
78	0.56	74.25
79	0.59	75.07
80	0.63	76.16
81	0.66	76.99
82	0.70	78.08
83	0.74	79.18
84	0.78	80.27
85	0.82	81.37
86	0.86	82.47
87	0.91	83.84
88	0.96	85.21
89	1.01	85.58
90	1.06	87.95
91	1.11	89.32
92	1.16	90.68
93	1.21	92.05
94	1.25	93.15
95	1.29	94.25
96	1.33	95.34
97	1.38	96.71
98	1.41	97.53
99	1.46	98.90
100	1.50	100

CASI = Cognitive Abilities Screening Instrument.

The percentage of participants with the lowest score was the respective dimensions were: 8.3% (long-term memory), 0.2% (orientation), 0.8% (abstraction and judgment), 9.1% (visual construction), and 9.7% (list-generating fluency). The other 4 dimensions had 0.0% of participants for the lowest score. There was no floor effect in the 9 dimensions. The percentage of participants with the highest score was the respective dimensions were: 26.5% (long-term memory), 1.0% (short-term memory), 5.7% (attention), 5.1% (mental manipulation), 10.3% (orientation), 1.2% (abstraction and judgment), 3.4% (language), 7.7% (visual construction), and 7.9% (list-generating fluency). Only the long-term memory dimension revealed a ceiling effect.

## 4. Discussion

This is the first study to use both the CFA and Rasch analysis to determine the unidimensionality of the CASI in people with dementia. The proposed one-factor model of the CASI fits well without any deletion processes in the CFA analysis. The unidimensionality of the CASI was supported based on the results of the Rasch analysis and residual variance in PCA. In a previous study, unidimensionality was examined using confirmatory factor analysis.^[10]^ Those results showed insufficient model fit of the CASI among normal adults and people with Alzheimer’s disease. The undimensionality of the CASI appears satisfactory in this study, indicating the 46 items measure a single construct. Thus, the score of each item can be summed up to reflect the general construct (e.g., overall cognitive function). For clinical implications, future users can apply the total score of the CASI to represent overall cognitive function, develop related treatment plans, and follow-up on overall cognitive function for people with dementia.

Acceptable person reliability of the CASI was confirmed, which implies that the overall cognitive function measured by the CASI in people with dementia is close to their real overall cognitive function. For each dimension, no floor or ceiling effect was shown, except in the long term memory dimension. Those without floor or ceiling effect indicate that these dimensions can discriminate the dimension-specific function for participants who have the lowest and highest scores. The long-term memory dimension showed obvious ceiling effect. One possible reason is that the items are easy general knowledge. Long-term memory permits persons to store information for long periods of time and these messages can be retrieved consciously (declarative memory) or unconsciously (non-declarative memory). Declarative memory contains 2 types: semantic memory (storing general knowledge) and episodic memory (storing personal experiences).^[31]^ To reduce the ceiling effect, future studies may consider designing more difficult general knowledge items and include items of personal experience.

The person-item map provided information regarding how well the items are distributed with regard to the ability of the respondent.^[24,25]^ Targeting results of 0.22 logits shows that the CASI 46 items had good targeting to the responders. In addition, The CASI assessed all participants similarly, independently gender. That is, the absence of DIF indicated that the CASI could be administrated to participants of different gender, without the concern that the items may mean something different to male or female.

To the best of our knowledge, this was the first study using Rasch analysis to examine the unidimensionality of the CASI. Rasch analysis allows transforming ordinal data to interval data which are crucial in both clinical and research settings. The transformed interval data of the CASI allowed clinicians to compare individuals or groups people with dementia on the same scale, which is not possible with ordinal data.^[32]^ In addition, the transformed interval data of the CASI could be used to track the true change across the scale continuum and to assess whether an intervention had led to an improvement in people with dementia.^[32,33]^ Based on the abovementioned, it is recommended to use transformed interval data of the CASI and a conversion table had been provided for future users.

The conversion table which shown the conversion from ordinal to interval-level data without modifying the original response of CASI could be useful for clinical purposes, as when consider reporting changes of the measure variable, an equal interval scaling allows to detect any variations.^[28]^ In addition, using this conversion table, the users do not need to conduct Rasch analysis every time to get the Rasch score when applying the CASI to assess cognitive function in people with dementia.

In the 6 dimensions (i.e., long-term memory, short-term memory, mental manipulation, orientation, abstraction and judgment, and language), some items belonging to their dimensions showed the same levels of item difficulty. For example, 2 items (i.e., general knowledge-new year and general knowledge-minute) in the long-term memory dimension appeared with the same level of item difficulty (−0.25). The same level of item difficulty may indicate item redundancy.^[34]^ The CASI takes about 20 minutes to administer.^[6]^ Future studies could design a computerized adaptive test with the CASI items, using fewer items to estimate the overall cognitive function for people with dementia.

Three limitations of this study are noted. First, the data in this study was collected from electronic medical records (secondary data). The quality of these data could be of concern. Future studies may conduct a prospective study to cross-validate our findings. Second, our data was collected in 1 hospital in northern Taiwan, which may restrict generalization. Third, we could only collect certain information from the electronic medical records (e.g., age, gender, diagnosis, CDR, and CASI) because of permissions. We could not identify other information in this study, such as onset of disease.

## 5. Conclusion

In this study, the 46 items of the CASI displayed a unidimensional construct. The CASI was able to capture overall cognitive function in people with dementia. Moreover, the CASI had acceptable personal reliability. Therefore, the CASI is a valid and reliable screening tool for assessing overall cognitive function in people with dementia.

## Acknowledgments

We would like to thank all the individuals who participated in this study.

## Author contributions

**Conceptualization:** En-Chi Chiu.

**Formal analysis:** Ya-Chen Lee, En-Chi Chiu.

**Investigation:** Shu-Chun Lee.

**Methodology:** Ya-Chen Lee, En-Chi Chiu.

**Resources:** Shu-Chun Lee.

**Supervision:** En-Chi Chiu.

**Validation:** Ya-Chen Lee, En-Chi Chiu.

**Writing – original draft:** Ya-Chen Lee, En-Chi Chiu.

**Writing – review & editing:** Ya-Chen Lee.
